# Implications of vestibular telemetry for the management of Ménière’s Disease—Our experience with three adults

**DOI:** 10.1111/coa.13676

**Published:** 2020-12-08

**Authors:** John S. Phillips, Jacob L. Newman, John E. FitzGerald, Stephen J. Cox

**Affiliations:** ^1^ Norfolk & Norwich University Hospitals NHS Foundation Trust Norwich UK; ^2^ University of East Anglia Norwich UK

**Keypoints**

- Diagnosis of the dizzy patient is challenging because the symptoms are momentary and assessment at presentation is often normal.
- The CAVA^®^ system continuously records 3‐axis head motions and both horizontal and vertical eye movements during a patient's normal daily activities (day and night), and importantly, during episodes of dizziness.
- Vestibular telemetry provided by the CAVA^®^ system can be used to differentiate between different causes of dizziness and to assist the clinician in formulating management options.
- Vestibular telemetry can clarify a patient's understanding of their own symptoms. This is important, as findings can vary greatly, despite a similar presentation of symptoms.
- Vestibular telemetry for individuals with a history of Ménière's disease can be very helpful in understanding the cause of recent episodes of dizziness and to formulate an appropriate treatment plan.

## 1 INTRODUCTION

Ménière's disease is a chronic idiopathic condition affecting the inner ear that results in repeated episodes of vertigo. The duration of a characteristic episode of vertigo is between twenty minutes and twelve hours according to contemporary classification systems.^1^, ^2^ Advancements in the field of vestibular telemetry have allowed the continuous ambulatory assessment of individuals with dizziness, vertigo and balance disturbance.^3^, ^4^ This article outlines the findings from assessing three patients with Ménière's disease and discusses how the use of vestibular telemetry aided their management.

## 2 MATERIALS AND METHODS

### 2.1 Trial design

This study was designed and conducted according to the Declaration of Helsinki of 1975, as revised in 1983, and was reviewed by the London ‐ Dulwich Research Ethics Committee (IRAS Number: 261 099). This report details the outcomes from preliminary trials using the CAVA® (Continuous Ambulatory Vestibular Assessment) system. This trial is an interventional clinical investigation to formally evaluate the capability of the CAVA^®^ system to detect pathological nystagmus.

### 2.2 Participants

Eligible participants were adults aged 18 or over, experiencing episodes of true vertigo, with at least two episodes within the preceding month. This report relates to three patients.

### 2.3 Interventions

**FIGURE 1:**
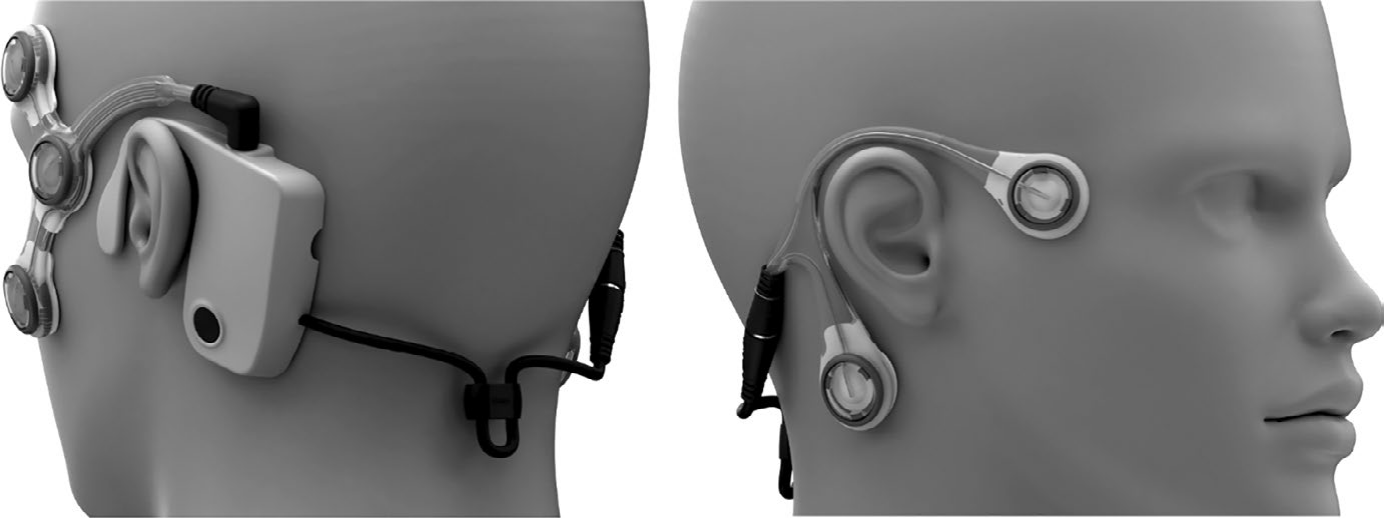
A depiction of the CAVA^®^ device when worn on the face. A reusable logging module sits behind the left ear, and two single‐use electrode mounts attach to the face. The mounts contain five ECG electrode pads, facilitating the capture of both horizontal and vertical eye movements. An “event marker” button on the logging module allows patients to log events of interest (eg the onset of dizziness). The device also captures head movement by way of a 3‐axis accelerometer

The CAVA^®^ system is composed of a piece of wearable technology, the CAVA^®^ device (Figure 1), plus the algorithms necessary to analyse the data it records. Detailed information regarding the CAVA^®^ system is available elsewhere,^3^, ^4^ but in essence, it allows the near‐continuous monitoring of eye and head movements of individuals experiencing dizziness for up to thirty days at a time. Parallels can be drawn between the CAVA^®^ device and the 24‐hour ECG tape that is used to identify cardiac arrhythmias^5^ and ambulatory EEG.^6^

Each patient wore the CAVA^®^ device during all of their normal daily activities, and also during the night, whilst sleeping. Patients were allotted an hour per day to remove the device for showering and to renew the device's electrodes. If patients experienced an episode of dizziness, they were instructed to press the device's event marker button, causing the device to record the date and time of the button press. Patients also maintained a written diary of any attacks experienced. At the end of the trial, the event marker data and diary records were used as a starting point from which to explore the eye‐movement traces for evidence of nystagmus. Once identified visually, a computer analysis highlighted candidate nystagmus beats in the signal and that output was then validated by a Consultant Clinical Scientist.

## 3 RESULTS

### 3.1 Patient one

**FIGURE 2:**
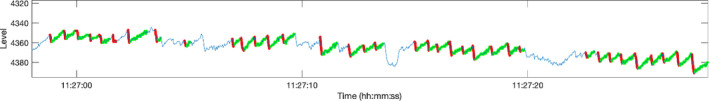
A 30‐second extract from the horizontal eye‐movement trace captured during a Ménière's attack reported by patient one. The trace shows clear evidence of left‐beating nystagmus. The nystagmus beats were automatically detected by a computer algorithm and then manually corrected by an expert

Patient one was a 53‐year‐old lady with a fifteen‐year history of left‐sided unilateral Ménière's disease. Discrete episodes of rotatory vertigo lasting for hours associated with severe nausea and aural fullness in the affected ear had been recently reported. Patient one wore the CAVA^®^ device and experienced no issues with the device after thirty consecutive days. Upon returning the device, the patient reported a single episode of vertigo lasting for approximately two hours. Assessing the data confirmed the presence of nystagmus—see Figure 2. The attack comprised eight periods of nystagmus, each lasting between approximately 21 and 460 seconds. Over the course of the attack, the nystagmus direction alternated from right‐beating to left‐beating, back to right‐beating, and finally to left‐beating. As this confirmed a true acute episode of vertigo, a discussion followed regarding the opportunities for intratympanic therapy.

### 3.2 Patient two

**FIGURE 3:**
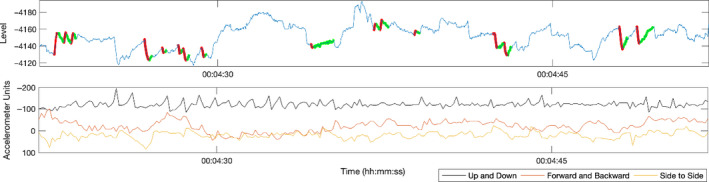
The top panel shows a 30‐second extract from the horizontal eye‐movement trace captured during an attack of dizziness reported by patient two. The trace shows short bursts of nystagmus which alternate between left‐ and right‐beating within the time period shown. The nystagmus beats were automatically detected by a computer algorithm and then manually corrected by an expert. The bottom panel shows the concurrent 3‐axis accelerometer data and reveals that the patient is moving their head during the dizzy attack

Patient two was a sixty‐year‐old man with a four‐year history of left‐sided unilateral Ménière's disease. He also reported distinct episodes of rotatory vertigo lasting for hours associated with severe nausea and aural fullness in the affected ear. Patient two also wore the CAVA^®^ device and experienced no issues with the device after thirty consecutive days. Upon returning the device, he reported many episodes of vertigo lasting for merely a few minutes at a time. Assessing the data confirmed nystagmus—see Figure 3. This trace is very different to that of patient one, as there were multiple short periods of nystagmus that often changed direction over a period of less than ten seconds. Typically, each burst lasted three seconds at most and contained two or three beats of nystagmus. Due to the nature of the nystagmus, it was considered that patient two was not experiencing typical acute episodes of vertigo as would be seen in a typical acute 'Ménière's attack'. The concurrent accelerometer data from the CAVA® device showed that the nystagmus was motion provoked, and therefore, suggestive of physiological compensation. As such, the patient was offered vestibular rehabilitation.

### 3.3 Patient three

**FIGURE 4:**
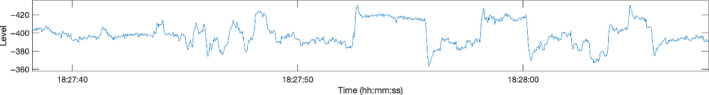
A 30‐second extract from the horizontal eye‐movement trace captured during an attack of dizziness reported by patient three. This trace shows no clear evidence of nystagmus and is representative of all other events indicated by the patient's trial diary and activation of the CAVA^®^ device‘s event marker

Patient three was a 39‐year‐old man with a one‐year history of left‐sided unilateral Ménière's disease. He reported distinct episodes of rotatory vertigo lasting for hours associated with severe nausea and aural fullness in the affected ear in a similar manner to the first two patients. Patient three wore the CAVA^®^ device and experienced no issues with the device after thirty consecutive days. Upon returning the device, he reported multiple episodes of almost daily vertigo lasting from between a few minutes to a few hours at a time. Assessing the data did not identify any episodes of nystagmus—see Figure 4. This trace is very different to that of patient one and patient two, as there were no definitive examples of nystagmus. When this was discussed with the patient, his history of symptoms evolved, whereby he provided a fuller and more detailed description of the history of his symptoms, and in doing this revealed that he does suffer with severe anxiety. He realised that it was possible he might actually be experiencing panic attacks at work rather than the true, severe and prolonged periods of vertigo that he had experienced in the past. Further questioning confirmed that these dizzy episodes were more in keeping with anxiety rather than with a disturbance of the vestibular system. As such, appropriate management of the patient's anxiety was arranged in collaboration with the patient's general practitioner.

## 4 DISCUSSION

### 4.1 Strengths of the study

The CAVA^®^ system has been developed to address the problem that individuals with dizziness, vertigo and balance disorders experience short‐lived episodic symptoms and present with little if any objective signs when they are assessed in a clinical environment. Parallels can be drawn between the CAVA^®^ system and technology employed in other clinical fields such as cardiology and neurology.^5^, ^6^

### 4.2 Synopsis of key findings

All three patients presented with similar symptoms but had very different findings. Of particular interest was how, when the system outputs were presented to each patient, they reported that they considered our explanation to support their ability to describe exactly what they were experiencing during an episode of apparent “vertigo.” For the two patients whose recorded attacks were not typical of Ménière's disease, this subsequently led to a discussion during which they revised the description of their dizziness experience. For the patient who did experience a typical attack, the data captured led to a detailed discussion with her regarding the timeline and features of their attack.

### 4.3 Clinical applicability of the study

In addition to clarifying patient symptoms, outputs from the CAVA® system are likely to provide further insight into the underlying mechanisms affecting the vestibular system for conditions such as Ménière's disease. From a fundamental clinical perspective, this would aid the identification of Ménière's disease and its subtypes, such as bilateral disease, to allow the correct determination of the active ear and thereby facilitate decisions regarding the appropriate treatment.

### 4.4 Comparisons with other studies

Much is unknown regarding the aetiology of Ménière's disease but having a tool to record the neurophysiological activity of the vestibular system during disease activity allows a unique opportunity to study this. Whilst there have been a number of isolated reports documenting eye movements of Ménière's disease patients during an acute attack of vertigo, these have been opportunistic ventures, and have only captured relatively small periods of nystagmus.^7^ More recently, video technology has been offered in the community in which goggles are supplied to patients with the instruction to wear them when a dizzy attack is anticipated.^8^ Young et al provide an impressive record of experience with such patients. However, it is not possible to wear the goggles continuously, and they do not record information prior to and after an attack. Furthermore, the use of videonystagmography (VNG) rather than electronystagmography (ENG) requires a patient who is just beginning to experience an acute vertigo attack to be able to gather their thoughts, remember where their goggles are stored, switch them on and then fit them correctly. The approach adopted for the CAVA® device, ENG, avoids these practical challenges and enables the device to be worn throughout the night. It also functions with the eyes open and closed.

### 4.5 Limitations of the study and further work

At this stage of our research program, the automatic detection of nystagmus in eye‐movement signals has been validated only for physiologically induced nystagmus in the horizontal plane.^3^, ^4^ Pathological nystagmus is quite different from the physiologically induced nystagmus analysed previously, as it is highly variable in terms of signal amplitude and frequency. Despite this, pathological nystagmus is clearly discernible in the data collected by the CAVA^®^ device. In view of this, a semi‐automatic approach was adopted here. Following this work and using the data presented, we are currently developing computer algorithms that use cutting‐edge Neural Network techniques to detect periods of pathological nystagmus, including nystagmus present in the vertical plane, such as that produced by patients with Benign Paroxysmal Positional Vertigo. Upon the completion of our current clinical investigation, we will formally evaluate our algorithm's accuracy at detecting these different forms of pathological nystagmus. Our long‐term goal is to develop reliable detection algorithms for pathological nystagmus to further allow the determination of aetiology via machine learning approaches.

## CONFLICT OF INTEREST

John Phillips takes full responsibility for the integrity of the content of this manuscript. Phillips, Newman and Cox are patent inventors for the mentioned product. There are no other competing interests to declare.

## AUTHOR CONTRIBUTIONS

JSP involved in project conception and design, data collection, analysis and write up. JLN involved in project design, data collection, analysis and write up. JEF involved in data analysis and write up. SJC involved in project conception and design, and write up.

## Data Availability

The data presented here are available upon reasonable request.

## References

[1] Goebel JA . 2015 Equilibrium Committee Amendment to the 1995 AAO‐HNS Guidelines for the Definition of Ménière's Disease. Otolaryngol Head Neck Surg. 2016;154(3):403‐404. 26884364 10.1177/0194599816628524

[2] Lopez‐Escamez JA , Carey J , Chung W‐H , et al. Diagnostic criteria for Ménière’s disease. J Vestib Res. 2015;25:1‐7. 25882471 10.3233/VES-150549

[3] Phillips JS , Newman JL , Cox SJ . An investigation into the diagnostic accuracy, reliability, acceptability and safety of a novel device for Continuous Ambulatory Vestibular Assessment (CAVA). Sci Rep. 2019;9(1):10452. 31320726 10.1038/s41598-019-46970-7 PMC6639326

[4] Newman JL , Phillips JS , Cox SJ . Automatic nystagmus detection and quantification in long‐term continuous eye‐movement data. Comput Biol Med. 2019;114:103448. 31577963 10.1016/j.compbiomed.2019.103448

[5] Mond HG . The Spectrum of Ambulatory Electrocardiographic Monitoring. Heart Lung Circ. 2017;26(11):1160‐1174. 28487061 10.1016/j.hlc.2017.02.034

[6] Dash D , Hernandez‐Ronquillo L , Moien‐Afshari F , Tellez‐Zenteno JF . Ambulatory EEG: a cost‐effective alternative to inpatient video‐EEG in adult patients. Epileptic Disord. 2012;14(3):290‐297. 22963900 10.1684/epd.2012.0529

[7] Watanabe TK .Nystagmus during an acute attack of Meniere's disease. ENGReport, pp. 1996; 1‐3.

[8] Young AS , Lechner C , Bradshaw AP , et al. Capturing acute vertigo: A vestibular event monitor. Neurology. 2019;92:e2743‐e2753. 31092626 10.1212/WNL.0000000000007644

